# The impact of polyunsaturated fatty acid-based dietary supplements on disease biomarkers in a metabolic syndrome/diabetes population

**DOI:** 10.1186/1476-511X-13-196

**Published:** 2014-12-16

**Authors:** Tammy C Lee, Priscilla Ivester, Austin G Hester, Susan Sergeant, Larry Douglas Case, Timothy Morgan, Ethel O Kouba, Floyd H Chilton

**Affiliations:** Department of Physiology/Pharmacology, Wake Forest School of Medicine, Medical Center Blvd, Winston-Salem, NC 27157 USA; Center for Botanical Lipids and Inflammatory Disease Prevention, Wake Forest School of Medicine, Medical Center Blvd, Winston-Salem, NC 27157 USA; Department of Urology, Wake Forest School of Medicine, Medical Center Blvd, Winston-Salem, NC 27157 USA; Department of Biochemistry, Wake Forest School of Medicine, Medical Center Blvd, Winston-Salem, NC 27157 USA; Division of Public Health Sciences, Department of Biostatistical Sciences, Wake Forest School of Medicine, Medical Center Blvd, Winston-Salem, NC 27157 USA; Center for Diabetes Research, Wake Forest School of Medicine, Medical Center Blvd, Winston-Salem, NC 27157 USA; Department of Internal Medicine/Molecular Medicine, Wake Forest School of Medicine, Medical Center Blvd, Winston-Salem, NC 27157 USA

**Keywords:** Diabetes, Metabolic syndrome, Polyunsaturated fatty acids (PUFAs), n-3 fatty acids, n-6 fatty acids, Eicosapentaenoic acid (EPA), Docosahexaenoic acid (DHA), Fish oil (FO), Botanical oil (BO; borage + echium)

## Abstract

**Background:**

Ingestion of polyunsaturated fatty acids (PUFAs) has been proposed to influence several chronic diseases including coronary heart disease (CHD) and type-2 diabetes (T2D).There is strong evidence that omega-3 (n-3) PUFAs provide protection against CHD and biomarkers of atherosclerosis. In contrast, there is more limited and inconsistent data for T2D. Few studies have examined the impact of n-3 PUFA-containing botanical oils on T2D.

**Methods:**

Fifty-nine subjects with early-stageT2D or metabolic syndrome participated in an 8-week, randomized, single-blind, parallel intervention study and were provided PUFA-containing oils. Individuals received either corn oil (CO), a botanical oil (BO) combination (borage [*Borago officinalis* L.]/echium oil [*Echium plantagineum* L.]) or fish oil (FO). The BO combination was enriched in alpha-linolenic, gamma-linolenic, and stearidonic acids and the FO in eicosapentaenoic and docosahexaenoic acids. Serum fatty acids and other serum lipids(triglycerides and total, HDL and LDL cholesterol), as well as markers of inflammation (leptin, and C-reactive protein) and glucose regulation (glucose and hemoglobin A1c) were assessed from fasting participants at baseline and after the intervention.

**Results:**

Compliance was verified by expected increases in specific PUFAs in each of the three oil arms. Participants in the CO group showed no differences in serum lipids, markers of inflammation or glucose regulation between pre- and post-treatment measures. Supplementation with BO significantly lowered total and LDL cholesterol levels and FO reduced serum triglycerides, hemoglobin A1c and increased HDL-cholesterol.

**Conclusion:**

Short-term dietary supplementation with BO and FO improved biomarkers associated with T2D/metabolic syndrome.

**Trial registration:**

Clinicaltrial.gov NCT01145066

## Background

Type 2 diabetes (T2D) is a major health concern in the United States with an estimated 18.3 million adults having physician-diagnosed diabetes, an additional 7.1 million adults having undiagnosed diabetes and about 81.5 million having prediabetes, also referred to as metabolic syndrome [1].The growing prevalence of obesity is strongly associated with low-grade systemic inflammation, dyslipidemia, insulin resistance and diabetes, and lifestyle factors including diet are known to play a critical role in the development and progression of T2D [2–4].

Consumption of oily fish, enriched in n-3 long chain polyunsaturated fatty acids (LcPUFAs) such as eicosapentaenoic (EPA) and docosahexaenoic acid (DHA), has long been observed to have health benefits [5, 6]. For example, consumption of these PUFAs has been shown to be effective in decreasing the risk of cardiovascular disease [7–10]. Based on these observations, it has been hypothesized that n-3 LcPUFAs may also have beneficial effects in T2D, glucose metabolism and insulin sensitivity. However, several recent meta-analyses and prospective studies [11–14] have revealed inconsistent results, and some studies even suggest that elevated n-3 intake may increase T2D in certain populations [15, 16]. Randomized clinical trials focused on the effects of PUFAs on the incidence of diabetes have not been conducted. However, several trials have examined whether T2D patients may benefit from n-3 LcPUFA supplementation [17, 18]; these too showed conflicting results.

**Figure 1:**
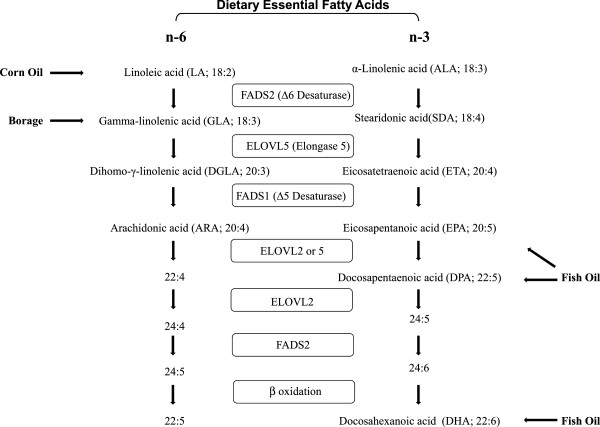
**Primary points at which PUFA-based supplements enter the PUFA biosynthetic pathway.** The biosynthesis of n-6 and n-3 PUFAs proceeds in parallel pathways utilizing the same set of fatty acid desaturase (FADS) and elongase (ELOVL) enzymes. Components of the dietary oils (Table 3) enter as both n-6 (LA, GLA) and n-3 (ALA, SDA, EPA, DPA, and DHA) PUFAs.

Inflammation is now thought to contribute to the development of a variety of chronic diseases, including T2D. There is a growing body of evidence in both human and animal studies that indicates that n-3 LcPUFAs inhibit inflammation by altering the transcription of key inflammatory genes and by competing with the n-6 LcPUFA, arachidonic acid (ARA), for key enzymes participating in pro-inflammatory eicosanoid biosynthesis (Figure 1) [19, 20]. Additionally, n-3 LcPUFAs can be converted to anti-inflammatory and pro-resolving molecules such as resolvins, protectins and n-3 derived endocannabanoids [21].

In addition to n-3 marine oils, botanical oils can be rich sources of n-6 and n-3 PUFAs. Figure 1 illustrates the primary points where oil-derived PUFAs enter the human PUFA biosynthetic pathway. Botanical oils have been observed to possess anti-inflammatory properties. Botanical oils such as borage and evening primrose were initially shown to block cyclooxygenase and lipoxygenase eicosanoid products and improve signs of rheumatoid arthritis [22–25]. Gamma-linolenic acid (GLA; 18:3 n-6) in evening primrose oil has also been shown to improve the course of mild diabetic neuropathy [26]. It has been shown that GLA is rapidly elongated to dihommo-GLA (DGLA) in humans, and this product and its cyclooxygenase metabolite, prostaglandin E_1_, has been proposed to attenuate inflammatory processes [24]. More recently, we have demonstrated that botanical oils derived from the seeds of the borage (*Borago officinalis* L.) and echium (*Echium plantagineum* L.) plants administered to asthmatic subjects effectively reduced the generation of pro-inflammatory ARA metabolites by circulating immune cells [27]. Borage oil, like evening primrose oil, is enriched with GLA, and echium oil contains alpha-linolenic (ALA) and stearidonic acids (SDA). We have also shown that this botanical oil combination elevates circulating LcPUFAs and improves glucose tolerance in insulin-resistant monkeys [28].

Together, these studies raise the question of whether botanical oil-based dietary supplements could impact T2D/metabolic syndrome in humans. Given the inconsistencies of the study designs and results derived from previously published studies with n-3 LcPUFAs and T2D and the potential impact of botanical PUFAs on T2D, we designed a randomized, single-blind, parallel intervention study to compare the impact of three PUFA-based supplements (corn oil, a botanical oil combination and fish oil) on levels of serum fatty acids and other serum lipids (triglycerides and total, HDL and LDL cholesterol), markers of inflammation (leptin, and C-reactive protein), as well as glucose regulation (glucose and hemoglobin A1c) in subjects with early-stage T2D and metabolic syndrome.

## Results

### Characteristics of the patient population

**Figure 2:**
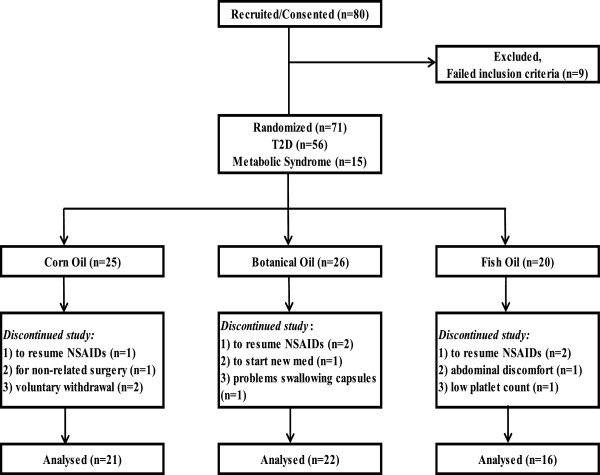
**Study design and randomization to intervention arms.** Eighty T2D or metabolic syndrome subjects were recruited into the double-blind, parallel intervention study and 71 were randomized to the three intervention arms. Fifty-nine subjects completed the study (74%). The reasons for withdrawals are shown for each arm.

Of the 80 participants entering the study, a total of 59 participants (74%) completed the study, with the remainder disqualifying (n = 9) or withdrawing (n = 12) for health reasons. As outlined in Figure 2, 59 participants were randomized to the three oil arms, receiving corn oil (CO), a botanical oil (BO; a combination of echium and borage oils), or fish oil (FO). The characteristics of this study population are detailed in Table 1. The uniformity of the baseline characteristics among the three arms was assessed by ANOVA single variable analysis. No significant differences in baseline characteristics among the arms were observed as calculated by Chi-square analysis or by One Way ANOVA.

**Table 1:** **Characteristics of the study population**

Valuable	Corn oil (CO)	Botanical oil (BO)*	Fish oil
	n (%)	n (%)	n (%)
No. of subjects	21 (35.6)	22 (37.3)	16 (27.1)
Female	15 (71.4)	10 (45.5)	10 (62.5)
African American	6 (28.6)	8 (36.4)	7 (43.8)
Diabetic	14 (66.7)	18 (81.8)	13 (81.3)
Metabolic syndrome	7 (33.3)	4 (81.2)	3 (18.8)
Hypertensive	16 (76.2)	19 (86.4)	9 (56.3)
	**Mean (SD)**	**Mean (SD)**	**Mean (SD)**
Age (years)	59.9 (9.8)	57.4 (7.8)	56.2 (8.7)
Weight (kg)	97.4 (17.2)	97.8 (18.7)	96.9 (15.1)
BMI (kg/m^2^)	34.8 (5.3)	34.1 (6.4)	33.2 (4.8)
Waist/hip ratio	0.9 (0.1)	0.9 (0.1)	0.9 (0.1)
Serum Glucose (mg/dl)	130.0 (44.9)	128.9 (50.8)	133.5 (64.7)

^*^BO arm received a combination of borage and echium oils.

p values were not significantly different between assignment groups, as calculated by Chi-square analysis of different proportions for each variable assigned to each treatment group, nor by One Way ANOVA for morphometric values [mean (SD)].

### Effect of supplementation on serum fatty acids

**Figure 3:**
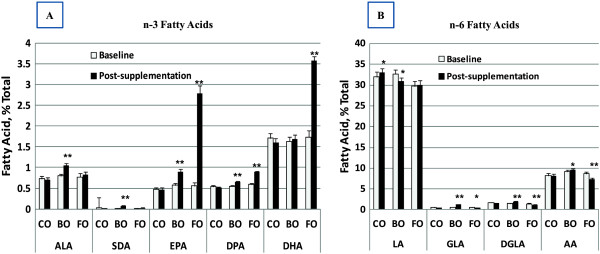
**Impact of dietary oil supplements on serum fatty PUFA profile.** Data are the mean (SD) at baseline (white bar) and after oil supplementation (black bar, combined 4- and 8-week data) for n-3 PUFAs **(A)** and n-6 PUFAs **(B)**. Pre – post changes within a supplementation arm that reached statistical significance are indicated by *, p < 0.05 and **, p <0.001.

The effects of dietary supplementation with CO, BO and FO on n-3 and n-6 PUFA levels are shown in Figure 3 and Table 2. Preliminary analyses demonstrated that the impact of supplementation on all fasting PUFA levels had stabilized by 4 weeks and did not change between the 4- and 8-week time points (data not shown). Thus, the data are expressed as baseline versus post-treatment supplementation levels of PUFAs (mean of combined 4- and 8-week data). Since CO contains little or no n-3 PUFAs (Table 3); it had no impact on circulating n-3 PUFA levels. Linoleic acid (LA, 18:2n-6) is the primary PUFA in CO, and there was a small increase in circulating LA levels after CO supplementation (Figure 3B).

**Table 2:** **Impact of dietary oil supplements on serum fatty acid**

Serum fatty acid profile	CO			BO			FO		
	Baseline ^*^ (SD)	Post-oil ^*^ (SD)	p-value	Baseline ^*^ (SD)	Post-oil ^*^ (SD)	p-value	Baseline ^*^ (SD)	Post-oil ^*^ (SD)	p-value
**Cl4:0**	0.70 (0.07)	0.66 (0.05)	0.56	0.69 (0.08)	0.66 (0.04)	0.86	0.72 (0.07)	0.62 (0.06)	0.78
**C14:1**	0	0		0	0		0	0	
**C15:0**	0.15 (0.02)	0.14 (0.02)	0.58	0.13 (0.02)	0.14 (0.02)	0.46	0.16 (0.02)	0.14 (0.02)	0.38
**C15:1**	0	0		0	0		0	0	
**C16:0**	21.19 (0.40)	20.75 (0.37)	0.23	20.64 (0.41)	20.49 (0.37)	0.71	21.65 (0.47)	20.76 (0.39)	0.51
**C16:1 n-7**	1.68 (0.13)	1.57 (0.13)	0.23	1.58 (0.08)	1.39 (0.08)	**0.03**	1.92 (0.24)	1.55 (0.14)	**0.002**
**C17:1**	0.08 (0.02)	0.04 (0.02)	0.24	0.10 (0.02)	0.05 (0.02)	**0.05**	0.13 (0.03)	0.06 (0.02)	**0.05**
**C18:0**	7.47 (0.09)	7.62 (0.12)	0.21	7.23 (0.14)	7.55 (0.16)	**<0.001**	7.00 (0.25)	7.39 (0.27)	**0.02**
**C18:1**	0.21 (0.06)	0.25 (0.05)	0.21	0.12 (0.07)	0.13 (0.05)	0.63	0.17 (0.06)	0.25 (0.06)	**0.02**
**C18:1 n-9**	20.63 (0.96)	20.30 (0.61)	0.15	20.22 (0.51)	19.60 (0.44)	0.10	21.92 (0.98)	20.24 (0.52)	**0.004**
**C18:1 n-11**	1.60 (0.06)	1.51 (0.06)	**0.03**	1.50 (0.07)	1.44 (0.05)	0.94	1.84 (0.06)	1.56 (0.06)	**<0.001**
**C18:2 n-6 [LA]**	31.99 (1.26)	33.11 (0.93)	**<0.001**	32.67 (0.98)	30.97 (0.80)	**0.002**	29.71 (1.28)	29.94 (1.14)	0.86
**C18:3 n-6 [GLA]**	0.53 (0.03)	0.52 (0.03)	0.32	0.59 (0.04)	1.20 (0.08)	**<0.001**	0.55 (0.05)	0.45 (0.05)	**0.01**
**C18:3 n-3 [ALA]**	0.73 (0.06)	0.71 (0.05)	0.85	0.80 (0.05)	1.05 (0.05)	**<0.001**	0.77 (0.09)	0.83 (0.06)	0.16
**C18:4 n-3 [SDA]**	0.03 (0.02)	0.01 (0.00)	0.82	0.01 (0.01)	0.08 (0.01)	**0.003**	0.01 (0.01)	0.02 (0.01)	0.69
**C20:0**	0.01 (0.01)	0.01 (0.00)	0.81	0.00 (0.00)	0.00) (0.00)	1.00	0.00 (0.00)	0.00 (0.00)	1.00
**C20:1 n-9**	0.08 (0.02)	0.05 (0.01)	0.09	0.04 (0.01)	0.04 (0.01)	0.83	0.10 (0.02)	0.03 (0.01)	**0.01**
**C20:2 n-6**	0.30 (0.02)	0.31 (0.01)	0.72	0.32 (0.01)	0.24 (0.01)	**<0.001**	0.33 (0.02)	0.23 (0.18)	**<0.001**
**C20:3 n-6 [DGLA]**	1.68 (0.08)	1.61 (0.05)	0.32	1.51 (0.08)	1.97 (0.09)	**<0.001**	1.43 (0.09)	1.45 (0.07)	**0.002**
**C20:4 n-6 [AA]**	8.17 (0.56)	8.11 (0.46)	0.83	9.12 (0.40)	9.16 (0.34)	**0.05**	8.68 (0.40)	7.31 (0.36)	**<0.001**
**C20:5 n-3 [EPA]**	0.48 (0.04)	0.46 (0.05)	0.28	0.573 (0.05)	0.89 (0.08)	**<0.001**	0.56 (0.07)	2.79 (0.18)	**<0.001**
**C22:0**	0	0		0	0		0	0	
**C22:1 n-9**	0.05 (0.04)	0.11 (0.04)	0.20	0.00 (0.00)	0.15 (0.06)	**0.008**	0.00 (0.00)	0.19 (0.11)	**0.03**
**C22:2 n-6**	0	0		0	0		0	0	
**C22:5 n-3 [DPA]**	0.54 (0.03)	0.51 (0.03)	0.25	0.55 (0.02)	0.65 (0.02)	**<0.001**	0.59 (0.03)	0.89 (0.03)	**<0.001**
**C24:0**	0.02 (0.02)	0.02 (0.01)	0.81	0.02 (0.02)	0.01 (0.01)	0.88	0.00 (0.00)	0.01 (0.01)	1.00
**C22:6 n-3 [DHA]**	1.71 (0.12)	1.60 (0.11)	0.13	1.63 (0.10)	1.58 (0.11)	0.56	1.73 (0.016)	3.58 (0.11)	**<0.001**
**C24:1 n-9**	0.03 (0.02)	0.01 (0.01)	0.25	0.01 (0.01)	0.00 (0.00)	0.50	0.04 (0.02)	0.00 (0.00)	0.25

*% total fatty acids.

Data are the mean (SD) at baseline and after oil supplementation (combined 4- and 8-week data). Pre – post changes within a supplementation arm that reached statistical significance are shown by a bolded p-value.

**Table 3:** **Fatty acid profile of the encapsulated oils and the daily dose of PUFAs consumed by each intervention arm**

	Fatty acid profile of dietary supplements (area%)					PUFAs provided in dietary supplements by arm (g/day)		
Common name	Fatty acid	Corn	Borage	Echium	Fish	CO	BO ^*^	FO
Myristic	C14:0	0	0	0	5.8			
Palmitic	C16:0	10.8	6.5	7.8	17.1			
Palmitoleic	C16:1 n-7	0	0	0.0	7.0			
Stearic	C18:0	2.8	3.3	5.0	4.1			
Oleic	C18:1 n-9	29.8	9.6	18.1	11.5			
Vaccenic	C18:1 n-11	0	0.4	0	3.0			
Linoleic (LA)	C18:2 n-6	54.2	22.1	18.6	1.9	3.96	1.80	0.48
Gamma-linoleic (GLA)	C18:3 n-6	0	41.0	9.7	0.3	0	1.65	0.05
Alpha-linoleic (ALA)	C18:3 n-3	0.8	0.2	28.0	1.2	0.06	1.90	0.23
Gondoic	C20:1 n-9	0.2	5.6	0	0.5			
Stearidonic (SDA)	C18:4 n-3	0.1	0	12.2	4.5	<0.01	0.83	0.86
Dihommo-γ-linolenic (DGLA)	C20:3 n-6	0	0	0	0.1			
Behenic	C22:0	0.1	0.3	0	1.3			
Arachidonic (ARA)	C20:4 n-6	0.2	0.1	0	0			
Erucic	C22:1 n-9	0	4.3	0.1	0			
Eicosopentaenoic (EPA)	C20:5 n-3	0.1	0	0	18.0	<0.01	0	3.58
Nervonic	C24:1 n-9	0	3.2	0.1	0.9			
Docosapentaenoic (DPA)	C22:5 n-3	0	0	0	2.1			
Docosahexaenoic (DHA)	C22:6 n-3	0	0	0	12.3	0	0	2.44
	Others	0.5	0.9	0	1.2			

^*^BO arm received a combination of borage and echium oils.

The fatty acid composition of the oils was evaluated by gas chromatography. The PUFA doses were calculated based on the mass of oil in the number capsules consumed daily, which were:9 CO capsules, 10 BO capsules (3 borage and 7 echium) or 9 FO capsules.

The BO provided alpha-linolenic acid (ALA, 18:3n-3) and stearidonic acid (SDA, 18:4n-3) from echium oil (Figure 1). Both of these PUFAs were significantly higher in the BO arm after supplementation (Figure 3A, Table 2) than at baseline. Eicosapentaenoic acid (EPA, 20:5n-3), the elongation and desaturation product of ALA and SDA, as well as EPA’s elongation product, docosapentaenoic acid (DPA, 22:5n-3), also rose after supplementation with the BO. However, there was no increase in circulating serum docosahexaenoic acid (DHA, 22:6n-3). The borage oil component of the BO supplement provided GLA (18:3n-6; Table 3). This n-6 PUFA is metabolized to its elongation and elongation/desaturation products, DGLA (20:3n-6) and ARA (20:4n-6), respectively; both were significantly higher after BO supplementation (Figure 3B).

As expected, FO supplementation induced a marked increase in serum levels of n-3 LcPUFAs including EPA, DPA and DHA. In contrast, FO also caused modest reductions in n-6 PUFAs including GLA, DGLA and ARA (Table 2). Overall, these data demonstrated that CO, BO and FO supplementation produced changes in serum PUFA levels that are consistent with the PUFA profile of the dietary oils. Additionally, these data show that the PUFAs in the supplements were clearly bioavailable and entered the PUFA biosynthetic pathway. Furthermore, it verifies that the subjects in each group were indeed compliant with capsule consumption.

### Effect of supplementation on disease biomarkers

The effects of dietary supplementation with CO, BO and FO on the serum lipid (triglycerides and total, HDL and LDL cholesterol) profile, markers of inflammation (leptin, and C-reactive protein) and glucose regulation (glucose and hemoglobin A1c) are shown in Table 4. Participants receiving CO did not experience significant changes in any of the disease biomarkers measured. In contrast, participants receiving BO showed a significant decrease in both total cholesterol (from 182.0 to 171.9 mg/dL; p = 0.05) and LDL cholesterol (from 106.3 to 96.8 mg/dL; p = 0.04). Participants receiving FO had significantly lower levels of triglycerides (from 187.2 to 156.8 mg/dL; p = 0.03), an increase in HDL-cholesterol (from 40.7 mg/dL to 43.6 mg/dL; p = 0.01) and a significant increase in insulin (from 19.1 μIU/mL to 24.6 μIU/mL; p = 0.02).

**Table 4:** **Impact of dietary oil supplements on serum lipid profile and biomarkers**

Arm	CO			BO			FO		
Variable	Baseline (SD)	Post-oil (SD)	p-value	Baseline (SD)	Post-oil (SD)	p-value	Baseline (SD)	Post-oil (SD)	p-value
T. Choles	183.0 (10.6)	176.8 (10.0)	0.25	182.0 (7.8)	171.9 (7.2)	**0.05**	180.6 (8.3)	183.0 (10.1)	0.71
HDL	46.0 (2.6)	46.3 (2.6)	0.73	47.6 (2.5)	48.6 (2.9)	0.29	40.7 (2.8)	43.6 (2.8)	**0.01**
LDL	102.7 (9.1)	98.4 (8.8)	0.53	106.3 (7.3)	96.8 (6.6)	**0.04**	102.6 (8.3)	107.9 (9.0)	0.32
TG^*^	172.9 (25.1)	158.4 (18.8)	0.38	140.2 (10.1)	132.0 (10.8)	0.14	187.2 (22.0)	156.8 (14.7)	**0.03**
CRP	2.36 (0.57)	3.72 (1.00)	0.41	3.03 (0.85)	3.06 (0.86)	0.99	6.08 (3.19)	3.59 (1.03)	0.19
Leptin^*^	53.5 (6.8)	44.2 (5.3)	0.38	33.3 (6.2)	33.9 (7.4)	0.82	45.1 (11.0)	40.6 (7.2)	0.37
Insulin	20.2 (3.2)	20.6 (3.3)	0.83	15.4 (2.0)	17.7 (2.6)	0.24	19.1 (4.5)	24.6 (6.8)	**0.02**
Glucose	130.0 (9.8)	130.7 (11.2)	0.93	128.9 (10.8)	125.9 (7.3)	0.93	133.5 (16.7)	128.7 (11.1)	0.33
HOMA-IR^*^	5.97 (0.83)	6.39 (1.08)	0.6	4.77 (0.84)	5.76 (1.09)	0.2	8.08 (2.76)	9.19 (3.03)	0.14
HbAlc	7.15 (0.34)	7.07 (0.35)	0.41	6.81 (0.30)	6.90 (0.27)	0.83	7.42 (0.33)	7.20 (0.32)	**0.05**

^*^p-values derived from log transformed data.

Data are the mean (SD) at baseline and after oil supplementation (combined 4- and 8-week data). Pre – post changes within a supplementation arm that reached statistical significance are shown by a bolded p-value.

As of 2013, the American Diabetes Association (ADA) criteria for the diagnosis of T2D included the presence of any one of the following: 1) hemoglobin A1c ≥ 6.5%; 2) fasting plasma glucose ≥ 126 mg/dL; 3) 2 hour plasma glucose ≥ 200 mg/dL following a 75 g oral glucose tolerance test; or 4) a random plasma glucose ≥ 200 mg/dL in a patient with symptoms of hyperglycemia or a hyperglycemic crisis [29]. Participants in all three supplementation arms had an average fasting serum glucose levels >126 mg/dL and an average hemoglobin A1c >6.5%. None of the oils had any impact on fasting glucose levels. However, there was a statistically significant reduction in hemoglobin A1c in the FO arm (p = 0.05).

C-reactive protein (CRP) is a non-specific marker of inflammation, but is often utilized as a tool in monitoring patients with diabetes and heart disease [30, 31]. Levels of CRP were measured at baseline and again post-treatment. Although none of the changes were statistically significant, there was a trend toward reduction in CRP levels (from 6.08 to 3.59 mg/L; p = 0.19) in the FO arm. There were no changes in any of the other disease biomarkers after supplementation with any of the oils (Table 4).

## Discussion

To date, randomized controlled and prospective population-based trials [32–35] that have assessed the impact of n-3 PUFAs on T2D and insulin sensitivity have provided inconsistent results regarding their effectiveness in preventing or treating T2D. More recent studies suggests that when an objective biomarker, such as levels of circulating LcPUFAs, are used as a measure of exposure (as opposed to dietary recall instruments), serum LcPUFAs are associated with long-term lower risk of T2D [36]. Given the uncertainty around this question and the fact that a very limited number of studies have examined the potential impact of BO supplementation in humans, we carried out a randomized, single blind, parallel intervention study to examine the impact of BO and FO on biomarkers associated with T2D. Specifically, this study was designed to examine the impact of CO, a BO combination (containing borage and echium oil) or FO on biomarkers of dyslipidemia, inflammation and glucose homeostasis.

A major consideration in this type of study is the choice of placebo. For example, in a recent meta-analysis of randomized controlled trials [37], placebos included CO, olive oil, palm oil, soybean oil and rapeseed oil. Each of these provides a challenge as a placebo because they contain potentially biologically active fatty acids such as linoleic acid (18:2n-6), oleic acid (18:1n-9) and erucic acid (22:1n-9), which may have their own biological effects. In the current study, we chose a parallel design to compare supplementation with three separate oils, CO, a BO combination or FO. While not a true placebo, we expected that CO would not impact biomarkers associated with T2D as it contains the most abundant PUFA (linoleic acid) found in the modern Western diet (MWD). Linoleic acid comprises 6 to 8% of energy in the MWD, so addition of ~4 g adds only marginally to daily LA exposure [38]. As expected, CO only marginally increased serum levels of LA and had no effect on levels of any other PUFAs. Likewise, CO had no impact on any of the biomarkers associated with T2D.

The key novel aspect of this study was the use of a BO combination composed of borage and echium oils, which contain both n-3 and n-6 PUFAs, and have been shown to influence disease biomarkers and clinical symptoms in several studies. For example, GLA-containing oils have been demonstrated to reduce rheumatoid arthritis disease activity and alter cytokine secretion and eicosanoid generation in numerous studies [22–25]. Echium oil, which contains SDA, has been shown to elevate n-3 LcPUFA levels, particularly EPA and reduce triglycerides in hypertriglyceridemic patients [39]. Echuim oil also has been shown to improve insulin sensitivity in non-human primates, and SDA has been proposed for the management and treatment of diabetes [28, 40]. Dietary consumption of milled flaxseed or flaxseed oil (containing ALA) was shown to have no impact on glycemic control [41]. We have recently determined the optimal ratios [27] of these two botanical oils that will elevate levels of anti-inflammatory PUFAs and reduce eicosanoids in asthmatic patients. However, we are not aware of randomized clinical trials that have specifically examined the impact of these botanical oils on T2D.

This BO combination induced increases in serum levels of several n-6 PUFAs including GLA and DGLA. As expected, there was also a small increase in circulating levels of ARA. This combination also elevated levels of n-3 PUFAs including ALA, SDA, EPA and DPA. With regard to biomarkers associated with T2D, the BO combination significantly impacted the lipid profile by reducing total and LDL cholesterol. There were also trends toward lower triglycerides and higher HDL cholesterol. There were no changes in glucose, CRP or leptin levels.

The most consistent effect of FO on disease biomarkers is its ability to lower triglycerides, especially in hypertriglyceridemic patients. N-3 LcPUFAs also have general anti-inflammatory properties. For example, DHA itself is known to directly inhibit NF-κB activation [42, 43], and DHA (and EPA)-derived resolvins and protectins [21, 44, 45] are proposed to dampen and resolve inflammatory responses. EPA also has important anti-inflammatory properties and effectively competes with AA for enzymes that participate in eicosanoid biosynthesis [46–48]. N-3 LcPUFAs also increase levels of n-3 derived endocannabinoids with potential anti-inflammatory effects [49]. As expected, supplementation with FO caused a marked elevation in serum n-3 LcPUFAs including EPA, DPA and DHA. Fish oil also markedly reduced triglycerides and induced an increase in HDL-cholesterol. Interestingly, hemoglobin A1c was significantly reduced in the FO arm, and there was a trend toward lower circulating glucose and CRP levels. However, it is important to point out that the FO arm began the study with higher baseline levels of CRP. There was also a small, but significant increase in insulin levels with FO supplementation. The explanation for this unexpected increase is unclear at this time.

There are a number of potential limitations to our study which include the length of the study (8 weeks), the placebo oil, the lack of control for background PUFA exposure, and the use of a patient population that contained both individuals with early-stage T2D and metabolic syndrome. We have addressed the placebo issue above and feel that CO is justified here as it adds only marginally to the PUFAs that are already being consumed in the background MWD. With regard to study length, serum PUFA levels reached equilibrium within 4 weeks of providing the supplements, and so there had been constant PUFA exposure to the subjects for at least 4 weeks. However, there have been some small reported effects of treatment duration on plasma lipids and lipoproteins, especially in studies of less than 3 months [49]. Additionally, some biomarkers such as hemoglobin A1c reflect cumulative changes that occur over several weeks to months, so treatment duration can be particularly important for these biomarkers. That being said, there was a significant decrease in hemoglobin A1c during the 8-week trial in the FO arm. It would be interesting to determine if FO further reduced this important biomarker during a longer period intervention. Another potential limitation of the study was the gastrointestinal symptoms observed in a few of the participants. However, these symptoms tended to be transient and only one patient chose to withdraw from the study because of this side effect. In terms of background PUFA exposure, the patients were provided a list of foods and supplements that contained high quantities of PUFAs not normally found in their diets and were told not to eat these foods or supplements during the study. Perhaps the best evidence for consistent PUFA exposure (between subjects) is the fact that aside from the PUFAs and their metabolites in supplements given, there was little individual variation in serum PUFAs from baseline levels (Table 2 and Figure 3).With regard to the patient population, there were no significant differences between the groups with regard to demographic variables listed in Table 1. Additionally, the groups have similar measures of glucose regulation including circulating glucose levels and hemoglobin A1c values. With these limitations taken into consideration, this study suggests that a BO combination, as well as FO, may have the capacity to reduce some risk factors associated with T2D/metabolic syndrome. Future studies utilizing longer interventions and larger sample sizes will be necessary to better understand the degree to which these supplements can impact dyslipidemia, inflammation and glucose homeostasis in patients with T2D and metabolic syndrome.

## Conclusion

This study has shown that short-term dietary supplementation with BO and FO has the capacity to improve disease biomarkers associated with T2D/metabolic syndrome. These results together with our previous observations that similar botanical oil combinations reduce eicosanoid generation in asthmatic patients [27] suggest that certain botanical oil combinations may have the capacity to improve a wide-range of disease biomarkers in inflammatory disorders.

## Methods

### Dietary oil supplements

Encapsulated oils obtained from echium seeds (*Echium plantagineum* L.), borage seeds (*Borago officinalis* L.), corn seeds (*Zea mays* L.) and fish (*Brevoortia tyrannis* Latrobe) were a generous gift from Croda Europe Ltd (Leek, Staffordshire, UK). The oils were authenticated by the Wake Forest School of Medicine Center for Botanical Lipids and Inflammatory Disease Prevention. Table 3 shows the fatty acid composition of each oil. Figure 1 illustrates the primary points where PUFAs in these oil supplements enter the human PUFA biosynthetic pathway.

### Subjects

The study was reviewed and approved by the Wake Forest University Health Sciences Institutional Review Board, a part of the Human Research Protection Program (HRPP). The inclusion criteria were: Adults aged ≥ 21 year, with either diabetes or metabolic syndrome. The study utilized The National Cholesterol Education Program Adult Treatment Panel (NCEP ATP) III definition of Metabolic syndrome requiring someone to have three or more of the five following risk factors: 1) central obesity as measured by waist circumference (men ≥ 40 inches; women ≥ 35 inches; 2) fasting blood triglycerides ≥ 150 mg/dL (or taking triglyceride-lowering medications); 3) low fasting blood HDL (men ≤ 40 mg/dL, women ≤ 50 mg/dL; 4) elevated blood pressure (≥130/85mmHG, or on blood pressure lowering medication); and 5) fasting glucose ≥ 100 mg/dL (or diabetes treatment) [50]. T2D was confirmed by documented history of treatment. Participant exclusion criteria included: 1) current use of anti-inflammatory drugs including NSAIDs, oral/IV steroids, other injection anti-inflammatory drugs (for rheumatoid arthritis), aspirin (>100 mg/day), leukotriene receptor antagonists, niacin, fibrates or fish oil; 2) blood pressure > 170/100 mm/Hg; 3) HbA1c > 10%; 3) fasting blood triglycerides > 500 mg/dL; 4) myocardial infarction, vascular surgery, or stroke in the past year; 5) any stage II/III/IV heart failure, prior cholecystectomy, or end stage renal disease ; 6) BMI <23 or > 45; 7) pregnancy; 8) alcohol use > 14 drinks per week; 9) self-reported current tobacco smoking or other illicit drug use; and 10) intolerance or allergy to fish oil.

Eighty (80) participants with either early-stage T2D or metabolic syndrome were recruited for the 8-week study (Figure 2). Early-stage T2D was defined as subjects with a diagnosis of T2D without evidence of end-stage organ damage secondary to their disease, including end-stage renal disease. Written informed consent was obtained from all subjects prior to enrollment. Nine of the initially recruited subjects were disqualified from the study because they failed to meet inclusion criteria.

### Study design

Consented participants were randomly assigned to one of three supplementation arms (Figure 2). Subjects in each arm consumed encapsulated oils along with their normal diets. The oils were: corn oil (CO);botanical oil (BO; combination of echium and borage oils); and fish oil (FO). These supplements were consumed with meals twice a day. The PUFA dosage in each oil arm is shown in Table 3 (right panel) and was achieved with the consumption of 9 daily CO capsules, 10 daily BO capsules (7 echium and 3 borage) or 9 daily FO capsules. The appearance of soft gelatin oil capsules was identical and participants were blind to the arm to which they were randomized. This was a single-blind study due to the differing number of daily capsules that were administered to each group. The ‘typical’ western diet provides very small quantities of gamma-linolenic acid (GLA) and stearidonic acid (SDA), limiting the ability to detect these PUFAs in circulating or cellular lipids. GLA and SDA are only typically observed when individuals are consuming GLA- or SDA-containing supplements such as borage, evening primrose, black currant, or echium oils. The use of any of these oils was an exclusion criterion of the study.

Participants were advised to minimize fish intake during the supplementation period, to refrain from self-medication with anti-inflammatory drugs and to inform the study staff about any health concerns during the study. The study was conducted in the Wake Forest University Health Sciences Clinical Research Unit where biospecimens (fasting blood and urine collected between 7:00 a.m. and 10:00 a.m.), vital signs (blood pressure and resting heart rate) and morphometric measurements (waist and hip circumference, height, body mass index [BMI] and body fat) were measured at baseline, 4- and 8-weeks after beginning supplementation.

Subjects reported any change in their medical condition at each study visit and used diary cards to record all medical symptoms and to log intake of study oils. Compliance was monitored by multiple mechanism including serum fatty acid profiles at 4- and 8-weeks, medication diaries and counts of returned capsules during the study. Capsule return counts indicated an average of 95% of capsules were taken (range = 76% - 100%).

The most common adverse events associated with taking PUFA-based dietary supplements were gastrointestinal symptoms reported as gas, constipation, loose stools, and/or abdominal pain or discomfort. These symptoms typically were mild, transient, occurred in the first few days of the study and were resolved within 2 to 3 days of first consuming the supplements.

A total of twelve (12) participants did not complete the study. One person dropped out of the study for abdominal discomfort and another from difficulty swallowing the capsules. Five participants withdrew from the study to resume taking non-steroidal anti-inflammatory drugs (NSAIDs); this was the most common reason for withdrawal. One participant withdrew due to an unrelated surgery and another needed to begin taking a new medication. One subject was removed from the study when their platelet count moved out of the normal range. Two subjects removed themselves from the study before 4-and 8-week samples could be acquired (Figure 2). There were no significant changes in vital signs in any study subjects during the study.

### Biochemical measurements

Measurements from blood samples included fasting glucose, insulin, high-sensitivity C-reactive protein (hs-CRP), leptin, hemoglobin A1c, CBC with differential/platelet, and serum lipids (total cholesterol, triglycerides, HDL-, VLDL- and LDL-cholesterol). Creatinine levels were obtained from urine samples. These endpoints were analyzed by a qualified clinical laboratory (Lab Corp, Burlington, NC).

### Fatty acid analysis

Serum was isolated from fasting whole blood and fatty acid methyl esters (FAME) were prepared [51] after saponification of duplicate samples (100 μl) in the presence of an internal standard (triheptadecanoin: Nuchek Prep, Elysian, MN, USA) as previously described [52]. A panel of 28 fatty acids was quantified by gas chromatography with flame ionization detection and individual fatty acids are expressed as percent of total fatty acids in each sample.

The fatty acid composition of the oil supplements (Table 3) was determined in aliquots of oil diluted in hexane and processed as described above. Individual fatty acids were expressed as percent of total fatty acids and as grams fatty acid/grams oil in order to calculate the fatty acid doses for each intervention arm.

### Statistical analysis

Baseline subject characteristics were summarized by the number (percent) for categorical data and mean (standard deviation) for continuous measures. Comparison of changes from baseline to the post supplemental period was performed by paired t-tests comparing the baseline value with the mean of the 4- and 8-week values. P values for the comparison of the cytokine levels were made using a logarithmic transformation because these values were skewed to the right and the transformation normalized the values and stabilized the variances. Measures of fatty acids were not normalized with a logarithmic transformation; therefore, non-parametric methods were used for hypothesis tests. The change in the distribution of fatty acid levels from pre- to post-treatment was compared using the sign-rank test and comparison of changes between oil supplementation arms were made by the Wilcoxon rank sum test.

## Abbreviations

ALA: Alpha-linolenic acid
ARA: Arachidonic acid
BMI: Body mass index
BO: Botanical oil (borage + echium oil combination)
hs-CRP: High sensitivity C-reactive protein
CHD: Coronary heart disease
CO: Corn oil
DGLA: Dihomo-gamma-linolenic acid
DHA: Docosahexaenoic acid
DPA: Docosapentaenoic acid
EPA: Eicosapentaenoic acid
FAME: Fatty acid methyl esters
FO: Fish oil
GLA: Gamma-linolenic acid
LA: Linoleic acid
Lc: Long chain
n-3: Omega-3
n-6: Omega-6
NCEP/ATPIII: National Cholesterol Education Program-Third Adult Treatment Panel
NSAID: Nonsteroidal anti-inflammatory drug
PUFA: Polyunsaturated fatty acid
SDA: Stearidonic acid
T2D: Type 2 diabetes.

## References

[1] Flegal KM, Carroll MD, Ogden CL, Curtin LR (2010). Prevalence and trends in obesity among US adults, 1999–2008. JAMA.

[2] Kopelman PG (2000). Obesity as a medical problem. Nature.

[3] Kahn SE, Hull RL, Utzschneider KM (2006). Mechanisms linking obesity to insulin resistance and type 2 diabetes. Nature.

[4] Boden G (2008). Obesity and free fatty acids. Endocrinol Metab Clin North Am.

[5] Bang HO, Dyerberg J, Sinclair HM (1980). The composition of the Eskimo food in north western Greenland. Am J Clin Nutr.

[6] Kromhout D, Bosschieter EB, de Lezenne CC (1985). The inverse relation between fish consumption and 20-year mortality from coronary heart disease. N Engl J Med.

[7] Harris WS (1997). n-3 fatty acids and serum lipoproteins: human studies. Am J Clin Nutr.

[8] Geleijnse JM, Giltay EJ, Grobbee DE, Donders AR, Kok FJ (2002). Blood pressure response to fish oil supplementation: metaregression analysis of randomized trials. J Hypertens.

[9] Balk EM, Lichtenstein AH, Chung M, Kupelnick B, Chew P, Lau J (2006). Effects of omega-3 fatty acids on serum markers of cardiovascular disease risk: a systematic review. Atherosclerosis.

[10] Lavie CJ, Milani RV, Mehra MR, Ventura HO (2009). Omega-3 polyunsaturated fatty acids and cardiovascular diseases. J Am Coll Cardiol.

[11] Strand E, Pedersen E, Svingen G, Schartum-Hansen H, Rebnord E, Bjorndal B, Seifert R, Bohov P, Meyer K, Hiltunen J, Nordrehaug J, Nilsen D, Berge R, Nygård O (2013). Dietary intake of n-3 long-chain polyunsaturated fatty acids and risk of myocardial infarction in coronary artery disease patients with or without diabetes mellitus: a prospective cohort study. BMC Medicine.

[12] Hartweg J, Farmer AJ, Perera R, Holman RR, Neil HAW (2007). Meta-analysis of the effects of n-3 polyunsaturated fatty acids on lipoproteins and other emerging lipid cardiovascular risk markers in patients with type 2 diabetes. Diabetologia.

[13] Stone NJ (1996). Fish consumption, fish oil, lipids, and coronary heart disease. Circulation.

[14] Saravanan P, Davidson NC, Schmidt EB, Calder PC (2010). Cardiovascular effects of marine omega-3 fatty acids. Lancet.

[15] Kaushik M, Mozaffarian D, Spiegelman D, Manson JE, Willett WC, Hu FB (2009). Long-chain omega-3 fatty acids, fish intake, and the risk of type 2 diabetes mellitus. Am J Clin Nutr.

[16] Djousse L, Gaziano JM, Buring JE, Lee IM (2011). Dietary omega-3 fatty acids and fish consumption and risk of type 2 diabetes. Am J Clin Nutr.

[17] Hartweg J, Farmer AJ, Holman RR, Neil A (2009). Potential impact of omega-3 treatment on cardiovascular disease in type 2 diabetes. Curr Opin Lipidol.

[18] Jeppesen C, Schiller K, Schulze MB (2013). Omega-3 and omega-6 fatty acids and type 2 diabetes. Curr Diab Rep.

[19] Calder PC (2009). Polyunsaturated fatty acids and inflammatory processes: New twists in an old tale. Biochimie.

[20] Calder PC (2002). Dietary modification of inflammation with lipids. Proc NutrSoc.

[21] Serhan CN, Gotlinger K, Hong S, Arita M (2004). Resolvins, docosatrienes, and neuroprotectins, novel omega-3-derived mediators, and their aspirin-triggered endogenous epimers: an overview of their protective roles in catabasis. Prostaglandins Other Lipid Mediat.

[22] Belch JJ, Hill A (2000). Evening primrose oil and borage oil in rheumatologic conditions. Am J Clin Nutr.

[23] Zurier RB, Rossetti RG, Jacobson EW, DeMarco DM, Liu NY, Temming JE, White BM, Laposata M (1996). gamma-Linolenic acid treatment of rheumatoid arthritis. A randomized, placebo-controlled trial. Arthritis Rheum.

[24] Johnson MM, Swan DD, Surette ME, Stegner J, Chilton T, Fonteh AN, Chilton FH (1997). Dietary supplementation with gamma-linolenic acid alters fatty acid content and eicosanoid production in healthy humans. J Nutr.

[25] Brzeski M, Madhok R, Capell HA (1991). Evening primrose oil in patients with rheumatoid arthritis and side-effects of non-steroidal anti-inflammatory drugs. Br J Rheumatol.

[26] Keen H, Payan J, Allawi J, Walker J, Jamal GA, Weir AI, Henderson LM, Bissessar EA, Watkins PJ, Sampson M, Gale E, Scarpello J, Boddie H, Hardy K, Thomas P, Misra P, Halonen J, The gamma-Linolenic Acid Multicenter Trial Group (1993). Treatment of diabetic neuropathy with gamma-linolenic acid. Diabetes Care.

[27] Arm JP, Boyce JA, Wang L, Chhay H, Zahid M, Patil V, Govindarajulu U, Ivester P, Weaver KL, Sergeant S, Israel E, Chilton FH (2013). Impact of botanical oils on polyunsaturated fatty acid metabolism and leukotriene generation in mild asthmatics. Lipids Health Dis.

[28] Kavanagh K, Flynn DM, Jenkins KA, Wilson MD, Chilton FH (2013). Stearidonic and gamma-linolenic acids in echium oil improves glucose disposal in insulin resistant monkeys. Prostaglandins Leukot Essent Fatty Acids.

[29] American Diabetes Association (2013). Standards of medical care in diabetes--2013. Diabetes Care.

[30] Mugabo Y, Li L, Renier G (2010). The connection between C-reactive protein (CRP) and diabetic vasculopathy. Focus on preclinical findings. Curr Diabetes Rev.

[31] Soinio M, Marniemi J, Laakso M, Lehto S, Ronnemaa T (2006). High-sensitivity C-reactive protein and coronary heart disease mortality in patients with type 2 diabetes: a 7-year follow-up study. Diabetes Care.

[32] Nettleton JA, Katz R (2005). n-3 long-chain polyunsaturated fatty acids in type 2 diabetes: A review. J Am Diet Assoc.

[33] Wang L, Folsom A, Zheng Z, Pankow J, Eckfeldt J (2003). Plasma fatty acid composition and incidence of diabetes in middle-aged adults: the Atherosclerosis Risk in Communities (ARIC) Study. Am J Clin Nutr.

[34] Vessby B, Aro A, Skarfors E, Berglund L, Salminen I, Lithell H (1994). The risk to develop NIDDM is related to the fatty acid composition of the serum cholesterol esters. Diabetes.

[35] Hu FB, van Dam RM, Liu S (2001). Diet and risk of Type II diabetes: the role of types of fat and carbohydrate. Diabetologia.

[36] Virtanen JK, Mursu J, Voutilainen S, Uusitupa M, Tuomainen TP (2014). Serum omega-3 polyunsaturated fatty acids and risk of incident type 2 diabetes in men: the Kuopio Ischemic Heart Disease Risk Factor study. Diabetes Care.

[37] Jafari T, Fallah AA, Azadbakht L (2013). Role of dietary n-3 polyunsaturated fatty acids in type 2 diabetes: a review of epidemiological and clinical studies. Maturitas.

[38] Blasbalg TL, Hibbeln JR, Ramsden CE, Majchrzak SF, Rawlings RR (2011). Changes in consumption of omega-3 and omega-6 fatty acids in the United States during the 20th century. Am J Clin Nutr.

[39] Surette ME, Edens M, Chilton FH, Tramposch KM (2004). Dietary echium oil increases plasma and neutrophil long-chain (n-3) fatty acids and lowers serum triacylglycerols in hypertriglyceridemic humans. J Nutr.

[40] Banz WJ, Davis JE, Clough RW, Cheatwood JL (2012). Stearidonic acid: is there a role in the prevention and management of type 2 diabetes mellitus?. J Nutr.

[41] Taylor CG, Noto AD, Stringer DM, Froese S, Malcolmson L (2010). Dietary milled flaxseed and flaxseed oil improve N-3 fatty acid status and do not affect glycemic control in individuals with well-controlled type 2 diabetes. J Am Coll Nutr.

[42] Komatsu W, Ishihara K, Murata M, Saito H, Shinohara K (2003). Docosahexaenoic acid suppresses nitric oxide production and inducible nitric oxide synthase expression in interferon-gamma plus lipopolysaccharide-stimulated murine macrophages by inhibiting the oxidative stress. Free Radic Biol Med.

[43] Spencer L, Mann C, Metcalfe M, Webb M, Pollard C, Spencer D, Berry D, Steward W, Dennison A (2009). The effect of omega-3 FAs on tumour angiogenesis and their therapeutic potential. Eur J Cancer.

[44] Serhan CN, Hong S, Gronert K, Colgan SP, Devchand PR, Mirick G, Moussignac RL (2002). Resolvins: a family of bioactive products of omega-3 fatty acid transformation circuits initiated by aspirin treatment that counter proinflammation signals. J Exp Med.

[45] Ariel A, Serhan CN (2007). Resolvins and protectins in the termination program of acute inflammation. Trends Immunol.

[46] Spector AA (1999). Essentiality of fatty acids. Lipids.

[47] Calder PC (2012). The role of marine omega-3 (n-3) fatty acids in inflammatory processes, atherosclerosis and plaque stability. Mol Nutr Food Res.

[48] Smith WL (2005). Cyclooxygenases, peroxide tone and the allure of fish oil. Curr Opin Cell Biol.

[49] Balvers MG, Verhoeckx KC, Bijlsma S, Rubingh CM, Meijerink J, Wortelboer HM, Witkamp RF (2012). Fish oil and inflammatory status alter the n-3 to n-6 balance of the endocannabinoid and oxylipin metabolomes in mouse plasma and tissues. Metabolomics.

[50] Grundy SM, Brewer HB, Cleeman JI, Smith SC, Lenfant C, Heart NHLBIA (2004). Definition of metabolic syndrome - Report of the National Heart, Lung, and Blood Institute/American Heart Association conference on scientific issues related to definition. Arteriosclerosis Thromb Vascul Biol.

[51] Metcalfe LD, Schmitz AA, Pelka JR (1966). Rapid Preparation of Fatty Acid Esters from Lipids for Gas Chromatographic Analysis. Anal Chem.

[52] Weaver KL, Ivester P, Seeds M, Case LD, Arm JP, Chilton FH (2009). Effect of dietary fatty acids on inflammatory gene expression in healthy humans. J Biol Chem.

